# Impact of Smoking on the Effectiveness of Advanced Therapies in Inflammatory Bowel Disease: Active Smoking Is Associated with Reduced Effectiveness of Anti-TNF Treatment

**DOI:** 10.3390/medicina62101833

**Published:** 2026-09-23

**Authors:** Sofia Cinque, Maria Laura Annunziata, Antonella Bianchi, Peter Bertoli, Giuseppe Dell’Anna, Monica Messina, Jacopo Fanizza, Orsola Raffaella Sociale, Monica Annese, Vito Annese

**Affiliations:** 1 Unit of Gastroenterology, IRCCS San Donato Policlinic, San Donato Milanese, 20097 Milan, Italy; sofia.cinque@grupposandonato.it (S.C.); marialaura.annunziata@grupposandonato.it (M.L.A.); antonella.bianchi2@grupposandonato.it (A.B.); peter.bertoli@grupposandonato.it (P.B.); dellanna.giuseppe@hsr.it (G.D.); monica.messina@grupposandonato.it (M.M.); jacopo.fanizza@grupposandonato.it (J.F.); orsolaraffaella.sociale@grupposandonato.it (O.R.S.); 2 Department of Gastroenterology, Faculty of Medicine, Vita-Salute University, San Raffaele, 20132 Milan, Italy; 3 Unit of Gastroenterology, IRCCS CSS Hospital, San Giovanni Rotondo, 71013 Foggia, Italy; annese.monica@gmail.com

**Keywords:** ulcerative colitis, Crohn’s disease, inflammatory bowel disease, smoking, anti-TNF, biologics, advanced therapy (AT)

## Abstract

*Background and Objectives*: Smoking is a well-established risk factor for Crohn’s disease (CD), whereas patients with ulcerative colitis (UC) who smoke may exhibit a milder disease course. The impact of smoking on the efficacy of advanced therapies (AT) in patients with inflammatory bowel disease (IBD) remains largely unknown. *Materials and Methods*: Patients with IBD receiving AT and with a detailed smoking history were retrospectively identified from our electronic database. The primary outcome was the rate of clinical response and remission. Secondary outcomes included endoscopic improvement, the need for AT sequencing, and surgery. *Results*: A total of 346 patients (136 UC and 210 CD) were analyzed, with a mean age of 47.3 years and a mean disease duration of 14.1 years (range, 0.5–48 years). Overall, 614 treatment courses were evaluated: 382 anti-TNF, 92 vedolizumab, 84 ustekinumab, 36 JAK inhibitor, 16 IL-23 inhibitor, and 4 etrasimod treatment courses. The anti-TNF effectiveness analysis included 371 infliximab/adalimumab courses after exclusion of 11 golimumab courses. A total of 107 patients (30.9%) were active smokers and 76 (21.9%) were former smokers. Anti-TNF therapy was significantly more effective in never smokers than in active smokers in the adjusted analysis (OR 1.44, 95% CI 1.04–2.00; *p* = 0.03), and this association remained significant in the mixed-effects model accounting for repeated treatment courses within patients (OR 1.35, 95% CI 1.01–1.80; *p* = 0.04). The association for advanced therapies overall was significant in the adjusted model (OR 1.25, 95% CI 1.01–1.55; *p* = 0.04) but was attenuated after accounting for repeated courses (OR 1.10, 95% CI 0.99–1.22; *p* = 0.07). No statistically significant differences were demonstrated for vedolizumab or ustekinumab, although the smaller sample sizes preclude exclusion of a clinically relevant effect. Former smokers had outcomes comparable to never smokers. Active smokers also required significantly more treatment courses than never smokers in both UC and CD (*p* = 0.03). *Conclusions*: Smoking is associated with a negative impact on the effectiveness of anti-TNF therapy in both UC and CD, highlighting the need for systematic smoking cessation counseling and patient education as an integral component of IBD management.

## 1. Introduction

Cigarette smoking is a major risk factor for numerous diseases, including cancer, cardiovascular disease, and chronic obstructive pulmonary disease [1]. In inflammatory bowel disease (IBD), namely ulcerative colitis (UC) and Crohn’s disease (CD), smoking is one of the most important and intriguing environmental factors contributing to disease pathogenesis. It has long been recognized that cigarette smoking is detrimental in CD, being associated with the development of a more severe disease phenotype, whereas it appears to exert a protective effect in UC [2,3,4,5,6,7]. The mechanisms by which tobacco smoking influences disease pathogenesis and disease course remain incompletely understood; however, proposed mechanisms include mucosal damage, impaired mucosal immune responses, compromised mucosal blood flow, and modulation of the gut microbiota through reduced bacterial diversity [8,9].

Cigarette smokers have an increased risk of developing CD (OR 1.76, 95% CI 1.40–2.22) [3,6], but a reduced risk of developing UC (OR 0.58, 95% CI 0.45–0.75) [3,10,11]. Whether these observational associations, which show opposite effects on the risk of developing CD and UC, reflect true causal relationships, confounding factors, or reverse causation remains uncertain [7].

Patients with established CD who smoke have an increased risk of disease relapse, hospitalization, and surgical intervention [12,13]. By contrast, patients who successfully maintain smoking cessation for more than 12 months experience a more benign disease course comparable to that of individuals who have never smoked [14]. Although patients with UC have a reduced risk of developing the disease, as discussed above, smoking status after diagnosis does not appear to influence disease course or clinical outcomes [3,10,11]. Most available studies, however, were conducted before or during the early years of the advanced therapy (AT) era. Therefore, although the extent to which immunosuppressive therapies can counteract the deleterious effects of smoking remains uncertain, tobacco use should be strongly discouraged, and smoking cessation support should be offered to all patients because of its well-established systemic adverse effects. Recently, the American College of Gastroenterology Clinical Guideline Update on preventive care in IBD strongly recommended that patients with IBD who smoke should be counseled to quit, also because of the potential detrimental effect of smoking on the development of colorectal cancer (CRC) [15]. Former smoking increased the risk of CRC in UC (HR 1.73, 95% CI 1.05–2.85), whereas passive smoke exposure showed no significant association. In CD, both active smoking (HR 2.20, 95% CI 1.02–4.76) and passive smoke exposure (HR 1.87, 95% CI 1.09–3.20) significantly increased the risk of CRC [16]. In addition, active smoking has been associated with the development of dermatological manifestations (in both UC and CD) and arthralgia (in CD) in patients with IBD receiving biologic therapy [17].

Over the last two decades, new smoking habits have emerged. Electronic cigarettes (e-cigarettes) deliver an aerosol (commonly referred to as vapor) by heating a solution that usually contains propylene glycol or glycerol, flavoring agents, and typically nicotine. More recently, heat-not-burn tobacco products (HNBTs), such as IQOS, have become available in several countries. Data regarding their impact on IBD outcomes remain scarce. Recently, their potential effect was investigated in patients with CD following surgery [18]. A total of 937 patients were included in this multicenter retrospective study, of whom 691 (74%) were nonsmokers, 176 (19%) conventional cigarette smokers, 37 (4%) HNBT users, and 33 (4%) e-cigarette users. Recurrence rates were significantly higher in all smoking groups than in nonsmokers (69.4%, 63.9%, and 60.6% for conventional cigarette smokers, HNBT users, and e-cigarette users, respectively, versus 40.8% for nonsmokers; all *p* < 0.05).

The introduction of biologics and small molecules has revolutionized the management of IBD by modifying disease progression and improving patients’ quality of life. Recent data have shown a progressive increase in the use of biologic therapies worldwide, with a substantial impact on healthcare expenditure [19]. Identifying factors that influence the effectiveness of advanced therapies is therefore crucial to support informed treatment decisions, optimize resource allocation, and improve patient care. However, the impact of smoking on the effectiveness of advanced therapies remains poorly understood.

Cigarette smoking increases the production of pro-inflammatory cytokines, including TNF-α and TNF receptors, while reducing the levels of anti-inflammatory cytokines [20,21]. In a recent meta-analysis [22], smoking was significantly associated with a reduced ability of infliximab or adalimumab to induce short-term clinical response and remission in patients with CD. Smokers treated with anti-TNF therapy were less likely to achieve a clinical response than nonsmokers (smokers defined as ≥5 cigarettes/day for ≥6 months: RR 0.63, 95% CI 0.48–0.83; lifetime never smokers versus ever smokers, excluding former smokers: RR 0.81, 95% CI 0.71–0.93). Current smokers were also less likely to achieve clinical remission in observational studies (RR 0.75, 95% CI 0.57–0.98), although this association was not observed in randomized controlled trials (RR 1.04, 95% CI 0.89–1.21).

One potential mechanism by which smoking may influence disease behavior, both in the general population and in patients with IBD, is through modulation of the gut immune system via alterations of the intestinal microbiota. Recently, Miyauchi et al. [23] provided new insights into how smoking may exert beneficial or detrimental effects in UC and CD, respectively. Individuals with UC who smoked exhibited increased fecal concentrations of short-chain fatty acids and aromatic compounds compared with those who had quit smoking, which may partly explain why smoking cessation is associated with disease exacerbation in some patients with UC.

In this study, we aimed to evaluate the impact of smoking on the effectiveness of advanced therapies in patients with both CD and UC. The primary outcome was treatment effectiveness in terms of clinical response and remission. Secondary outcomes included the need for treatment sequencing, surgery, endoscopic remission/response, and normalization of inflammatory biomarkers (C-reactive protein [CRP] and fecal calprotectin [FC]).

## 2. Materials and Methods

### 2.1. Patients

Patients with IBD receiving AT and with a detailed smoking history were retrospectively identified from the electronic database of the IBD Referral Center at IRCCS Policlinico San Donato, San Donato Milanese, Milan, Italy, from 1999 onward. For the purposes of this study, the database was frozen on 30 June 2026. A total of 372 patients who lacked reliable information on smoking habits or had never received advanced therapy were excluded (Figure 1). Demographic characteristics, clinical variables, and outcome data were recorded. The demographic and clinical characteristics of the study population are summarized in Table 1.

### 2.2. Treatment

All patients who received at least 8 weeks of treatment with infliximab (including biosimilars), adalimumab (including biosimilars), golimumab, vedolizumab, ustekinumab (including biosimilars), tofacitinib, filgotinib, upadacitinib, mirikizumab, risankizumab, guselkumab, or etrasimod were included. Dose and administration schedules followed the approved standard regimens; however, treatment intensification was allowed at the discretion of the treating physician in cases of loss of response.

### 2.3. Evaluation of Efficacy

Treatment efficacy was evaluated for each treatment course. Clinical remission was defined according to the STRIDE II recommendations [24]. For CD, clinical remission was defined as a Crohn’s Disease Activity Index (CDAI) < 150 or a Harvey–Bradshaw Index (HBI) < 5, whereas clinical response was defined as a reduction of at least 100 points in the CDAI or at least 3 points in the HBI. For UC, clinical remission was defined as a partial Mayo (pMayo) score < 3, with no individual subscore > 1. Clinical response was defined as a reduction of at least 30% in the Mayo score. Efficacy was not assessed at a fixed time point; instead, each treatment course was evaluated at the last available follow-up after completion of at least the induction phase (8 weeks), except when treatment was discontinued because of an infusion reaction.

Treatment intolerance was defined as the occurrence of adverse events leading to treatment discontinuation or switching. Primary failure was defined as the absence of clinical response or worsening of symptoms during the induction phase. Loss of response (LOR) was defined as recurrence of symptoms following an initial response or remission, leading to treatment switching despite therapy optimization.

Endoscopic remission in CD was defined as the absence of ulcers or a Rutgeerts score of i0 or i1 following surgery, whereas endoscopic response was defined as an improvement of more than 50% in the SES-CD or an improvement of at least one point in the Rutgeerts score. In UC, endoscopic remission was defined as a Mayo endoscopic subscore of 0, whereas endoscopic response was defined as a reduction of at least one point in the Mayo endoscopic subscore. Endoscopic remission and endoscopic response were assessed at the last available follow-up. Similarly, normalization of C-reactive protein (CRP) and fecal calprotectin (FC) was evaluated at the last available follow-up.

### 2.4. Impact of Smoking

Smoking habits were assessed using a standardized questionnaire and updated at each consultation, which occurred at least twice annually. A final check was done by telephone interview during May–June 2026 for patients who had no follow-up visit during 2026. Current smokers were defined as individuals who smoked at least one cigarette per day according to the World Health Organization definition. The same definition was also applied to e-cigarette smokers. For both current and former smokers, information regarding smoking duration, time since smoking cessation, number of cigarettes smoked per day (or, where applicable, cigar or pipe use), and use of e-cigarettes was collected. Passive smoking exposure was not evaluated because it was considered difficult to quantify accurately.

### 2.5. Statistical Analysis

Descriptive and inferential statistical methods were used to ensure a robust analysis. Descriptive statistics included mean and standard deviation for normally distributed continuous variables (e.g., age), median and interquartile range for non-normally distributed variables, and frequencies and percentages for categorical variables (e.g., sex and smoking status). Comparisons between groups were performed using Student’s *t*-test or the Mann–Whitney U test, depending on the data distribution, for continuous variables, and the chi-square test or Fisher’s exact test for categorical variables. Multivariable logistic regression analysis was performed to evaluate the association between smoking status and binary outcomes (e.g., response versus no response to therapy), after adjustment for potential confounding variables. Covariates included in the model were therapy type, disease duration, disease type (UC, CD, IBD), and surgery. In addition, the analyses were repeated using a mixed-effects logistic regression including patient identifier as a random effect. Concomitant use of steroid and immunomodulators was not included because treatment duration could not be accurately determined owing to the retrospective nature of the study.

## 3. Results

Table 1 summarizes the characteristics of the study population, including disease location and behavior according to the Montreal classification. Active smokers were significantly more frequent among patients with CD than among those with UC (30% vs. 19.8%; *p* = 0.04), whereas perianal disease and surgery during follow-up were significantly more common in patients with CD (*p* < 0.0001). The cohort was predominantly Italian and Caucasian, which may limit the generalizability of the findings to more ethnically diverse populations. Consistent, detailed information on comorbidities and related concomitant therapies was not available in the database, apart from extraintestinal manifestations.

Table 2 summarizes the advanced therapies evaluated, comprising 249 treatment courses in 136 patients with UC and 365 treatment courses in 210 patients with CD. Overall, 79 patients (23%) received more than two courses of advanced therapy, reflecting the characteristics of a difficult-to-treat cohort referred to a tertiary IBD center with a long duration of follow-up (mean 14.1 years; range, 0.5–48 years). Of note, given the standard regulation in Italy, all patients had received conventional therapy before starting AT with mesalamine (all UC and 64 CD patients), corticosteroids (114 UC and 170 CD), azathioprine (47 UC and 75 CD), and methotrexate (2 UC and 7 CD).

In addition, because of national regulation, virtually all patients received infliximab, adalimumab, or their biosimilars as first-line advanced therapy. Other biologics, and subsequently JAK inhibitors, could generally be used only in cases of infusion reactions or treatment failure. Only three patients received vedolizumab as first-line therapy because of a recent history of cancer, and four received ustekinumab because of concomitant psoriasis. Dose optimization for loss of response was used in approximately 20–30% of patients but was not systematically tracked in the database because different strategies were employed (higher doses, shorter intervals for variable periods, or re-induction).

Table 3 summarizes the effectiveness of the different advanced therapies in terms of clinical outcomes (clinical remission or response). Treatment intolerance leading to discontinuation or switching occurred in 9.4% of treatment courses (58/614), mainly because of infusion reactions to intravenous infliximab. Primary treatment failure occurred in 20% of anti-TNF treatment courses with infliximab and adalimumab (whereas failure occurred in 8 of 11 golimumab courses for unexplained reasons), 26% of vedolizumab courses, 14% of ustekinumab courses, and 11% of JAK inhibitor courses. Loss of response leading to treatment switching occurred in 12%, 11%, and 19% of treatment courses with anti-TNF therapy, vedolizumab, and ustekinumab, respectively. Data regarding the effectiveness of JAK inhibitors, IL-23 inhibitors, and etrasimod should be interpreted with caution because patients receiving these newer agents had relatively short follow-up.

Table 4 reports the primary outcome of the study. For each therapy, the number of treatment courses in both UC and CD is reported, and clinical outcomes are categorized as intolerance, clinical response (including remission), primary failure, or loss of response according to smoking status. Patients who discontinued treatment because of intolerance were excluded from the analysis evaluating the impact of smoking on treatment effectiveness.

Overall (Table 4a), smokers with IBD had a significantly lower response rate to advanced therapies than never smokers (54.5% vs. 67.5%; *p* = 0.006), whereas former smokers had a response rate comparable to that of never smokers (70.8% vs. 67.5%). A similar trend was observed in both UC and CD, with smokers achieving lower response rates than never smokers (UC: 51.7% vs. 64.2%, *p* = 0.05; CD: 56% vs. 70.5%, *p* = 0.01). No significant differences were observed between light smokers (≤10 cigarettes/day), moderate smokers (11–20 cigarettes/day), and heavy smokers (>20 cigarettes/day), although the number of patients in these subgroups was limited. Similarly, no significant association was observed between cumulative smoking exposure (pack-years) and treatment response, although this analysis was also limited by the small sample size. No significant differences were observed among exclusive e-cigarette users (*n* = 15); of note, two additional subjects used both e-cigarettes and conventional cigarettes. All these patients were analyzed together with conventional cigarette smokers. Regarding former smokers, information about the duration of quitting was available only in 26 of 76 patients and the large majority (20 subjects) had stopped smoking more than 5 years at the time of starting AT.

We next evaluated the effectiveness of each advanced therapy according to smoking status. The analysis of anti-TNF therapy (Table 4b) combined infliximab and adalimumab (excluding golimumab because of the small sample size and unusually high failure rate) because these agents share the same therapeutic target and the sample size for each individual drug was insufficient to allow adequately powered subgroup analyses. The response rate was significantly lower in smokers (51%) than in never smokers (67%; *p* = 0.01), both in CD (56% vs. 72%; *p* = 0.03) and UC (36% vs. 59%; *p* = 0.04). Notably, no significant differences were observed between former smokers and never smokers.

When infliximab and adalimumab were analyzed separately, the same trend was observed; however, the differences did not reach statistical significance, most likely because of the insufficient statistical power. For infliximab, response rates in smokers versus never smokers were 55% versus 69% (*p* = 0.08), 63% versus 73% (*p* = 0.20), and 44% versus 63% (*p* = 0.15) in the overall IBD cohort, CD, and UC, respectively. For adalimumab, the corresponding response rates were 47% versus 63% (*p* = 0.055), 52% versus 70% (*p* = 0.06), and 14% versus 52% (*p* = 0.08), respectively.

No significant differences were observed between smokers and never smokers receiving vedolizumab (63% vs. 59%; crude OR for response in never versus active smokers 0.84, 95% CI 0.32–2.17), while a numerical difference was seen for ustekinumab (58% vs. 73%; crude OR 1.97, 95% CI 0.74–5.26) (Table 4c, Figure 2). Because of the limited sample size and short duration of follow-up, no definitive conclusions can be drawn regarding the impact of smoking on these therapeutic classes.

**Figure 2:**
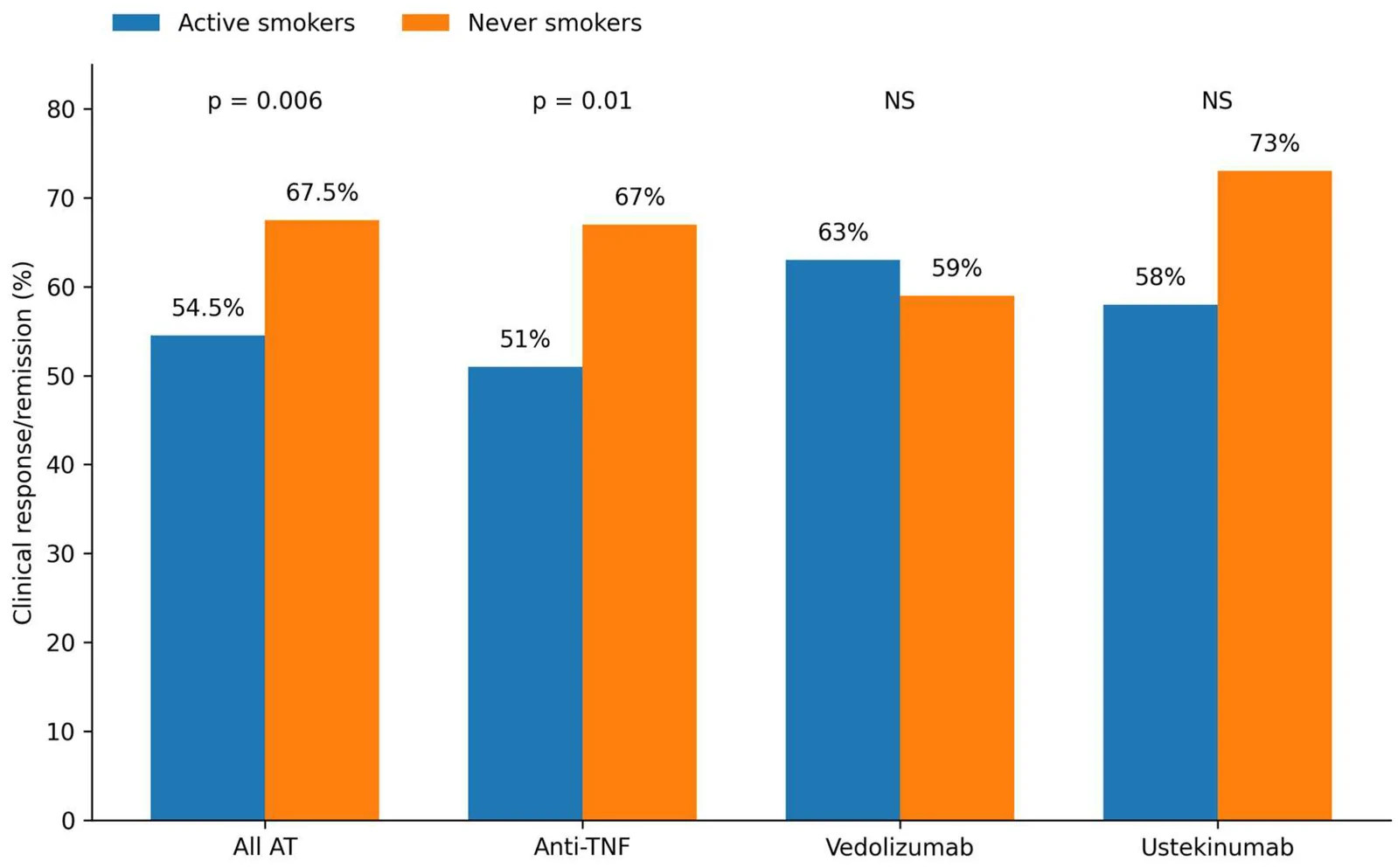
Clinical response/remission rates according to smoking status and drug class. Active smokers had lower response rates than never smokers for all advanced therapies (54.5% vs. 67.5%; *p* = 0.006) and anti-TNF therapy (51% vs. 67%; *p* = 0.01). No statistically significant difference was observed for vedolizumab (63% vs. 59%) or ustekinumab (58% vs. 73%); these subgroup analyses were limited by sample size.

We subsequently evaluated the secondary outcomes, specifically the number of advanced therapy courses required during follow-up. As shown in Table 5, active smokers, former smokers, and never smokers had comparable ages at diagnosis, disease duration and follow-up duration in both UC and CD. However, compared with never smokers, active smokers more frequently required two or more courses of advanced therapy, both in UC (73.5% vs. 52.2%; *p* = 0.03) and CD (65.8% vs. 48.9%; *p* = 0.03). No significant differences were observed between former smokers and either active or never smokers. In addition, patients with CD who were active or former smokers underwent significantly more surgical procedures than never smokers (45.2% and 48.9%, respectively, vs. 32.6%; *p* = 0.04).

Finally, we evaluated endoscopic remission and normalization of inflammatory biomarkers at the last available follow-up by comparing active smokers with never smokers.

Endoscopic remission within 2 years before the analysis was available in 282 out of 346 patients. Endoscopic remission was found in 125 patients, 49 UC and 76 CD. Among UC patients with endoscopic data and known smoking status, remission was observed in 17/29 (58.6%) active smokers and 22/61 (36.1%) never smokers. Among CD patients, remission was observed in 21/57 (36.8%) active smokers and 39/81 (48.1%) never smokers. Thus, the numerical direction differed between UC and CD. These differences were not statistically significant and the analyses were underpowered; they should therefore be considered exploratory.

The last available inflammatory biomarker assessment was retrieved for patients with available CRP and/or fecal calprotectin measurements. Biomarker normalization was defined as normalization of the available marker (CRP 5 mg/L and/or fecal calprotectin 200 µg/g); both biomarkers were not necessarily available in all patients. Among patients with UC, normalization of the available biomarker was observed in 25/30 (83.3%) active smokers and 30/61 (49.2%) never smokers. Among patients with CD, the corresponding proportions were 32/58 (55.2%) and 38/86 (44.2%), respectively. Biomarker measurements represented the last available assessment and were not obtained at a prespecified time point or necessarily concomitantly with the final clinical or endoscopic assessment. These findings should therefore be regarded as exploratory.

A multivariable logistic regression analysis was performed to further evaluate the independent association between smoking and the effectiveness of advanced therapies while adjusting for potential confounding variables (Table 6). Never smokers had a significantly higher likelihood of responding to advanced therapy than active smokers in the adjusted model (OR 1.25, 95% CI 1.01–1.55; *p* = 0.04). The association was also significant for anti-TNF therapy (OR 1.44, 95% CI 1.04–2.00; *p* = 0.03). To account for repeated treatment courses within the same patient, a mixed-effects logistic regression including patient identifier as a random effect was performed. In this model, the association between smoking status and anti-TNF effectiveness remained statistically significant (OR 1.35, 95% CI 1.01–1.80; *p* = 0.04), whereas the association considering all advanced therapies was attenuated and no longer statistically significant (OR 1.10, 95% CI 0.99–1.22; *p* = 0.07).

## 4. Discussion

As the prevalence of IBD continues to rise worldwide, so does its economic burden on healthcare systems. The increasing use of biologics and small molecules has substantially increased the overall cost of IBD care, with medications now representing the major cost driver [25]. An updated European inception cohort (2010–2015) reported that 75% of patients with CD and 50% of those with UC received biologic therapy within five years of diagnosis [26]. Despite the expanding therapeutic armamentarium, reliable predictors of response to advanced therapies (AT) remain limited. By contrast, relatively little attention has been paid to potentially modifiable environmental factors, such as cigarette smoking, that may influence treatment outcomes.

In this single-center retrospective longitudinal study, we evaluated the association between active smoking and the effectiveness of advanced therapies, including biologics and small molecules, in patients with IBD. To our knowledge, this is the first study to evaluate the impact of smoking on treatment effectiveness in both CD and UC. We analyzed a large cohort comprising 210 patients with CD and 136 with UC, with detailed information on smoking habits collected over a mean follow-up of 14 years. Overall, 614 treatment courses were evaluated, of which 62% involved anti-TNF therapy. The main findings of the study can be summarized as follows:

(a) Anti-TNF therapy was significantly more effective in never smokers than in active smokers in the adjusted analysis (OR 1.44, 95% CI 1.04–2.00; *p* = 0.03), and the association remained significant after accounting for repeated treatment courses within patients (OR 1.35, 95% CI 1.01–1.80; *p* = 0.04).
(b) Advanced therapies overall were more effective in never smokers than in active smokers in the adjusted analysis (OR 1.25, 95% CI 1.01–1.55; *p* = 0.04), although the association was attenuated and no longer statistically significant after accounting for repeated treatment courses within patients (OR 1.10, 95% CI 0.99–1.22; *p* = 0.07).
(c) Compared with never smokers, active smokers more frequently required two or more additional courses of advanced therapy in both UC (73.5% vs. 52.2%; *p* = 0.03) and CD (65.8% vs. 48.9%; *p* = 0.03), despite a comparable duration of follow-up.
(d) Former smokers achieved treatment outcomes comparable to those of never smokers; however, cessation duration was available in only 26 of 76 former smokers, limiting interpretation of this finding.

These findings have important clinical implications and further reinforce the need for systematic smoking cessation counseling as an integral component of IBD management. The information available in our database does not allow us to determine how long smoking cessation is required to reverse the potential negative influence of smoking; however, among patients with available data, most had stopped smoking more than five years before starting AT. By contrast, no definitive conclusions can be drawn regarding the influence of smoking on the effectiveness of other biologics and small molecules because of the limited number of patients treated with these therapeutic classes and their relatively short duration of follow-up.

The apparent discordance between clinical, endoscopic, and biomarker outcomes should be interpreted cautiously because these assessments were not performed at standardized or necessarily coincident time points. Moreover, treatment effectiveness was evaluated across individual treatment courses, whereas endoscopic and biomarker outcomes represented patient-level assessments at the last available evaluation; these endpoints therefore capture different dimensions and time points of the disease course and should not be expected to be directly concordant.

The mechanisms underlying the association between cigarette smoking and reduced anti-TNF effectiveness are likely multifactorial. Cigarette smoke contains thousands of bioactive compounds capable of inducing oxidative stress, endothelial dysfunction, and activation of both innate and adaptive immune responses. Experimental studies have consistently shown that smoking promotes the production of pro-inflammatory cytokines, including TNF-α, IL-1β, and IL-6, enhances NF-κB activation, alters macrophage polarization toward a pro-inflammatory phenotype, disrupts epithelial barrier function, and induces profound changes in the intestinal microbiota [23,27,28]. Collectively, these alterations may sustain intestinal inflammation despite pharmacological TNF blockade, thereby reducing the effectiveness of anti-TNF therapy, as suggested by earlier experimental and clinical studies [29,30,31].

Our findings are consistent with the growing body of evidence indicating that smoking adversely affects the effectiveness of anti-TNF therapy in Crohn’s disease. A recent systematic review by Berkowitz and colleagues [8] concluded that the available evidence supports reduced anti-TNF effectiveness among smokers, although most published studies were limited to CD and data on newer biologic agents remain scarce. By contrast, evidence in ulcerative colitis is virtually absent. Our findings therefore extend the current literature by suggesting that the detrimental association between smoking and anti-TNF therapy is not confined to CD but may also be clinically relevant in UC.

Several pharmacokinetic and immunological mechanisms may account for this association. Although therapeutic drug monitoring was not available in our cohort, previous studies have suggested that smokers receiving infliximab may have lower circulating drug concentrations and an increased propensity to develop anti-drug antibodies [32]. Persistent systemic inflammation may increase drug clearance, thereby reducing effective drug exposure. Whether similar mechanisms apply to adalimumab remains uncertain; nevertheless, these observations provide a biologically plausible explanation for our findings. Future prospective studies incorporating therapeutic drug monitoring and assessment of immunogenicity are warranted to clarify these mechanisms.

The observation that smoking was associated with reduced effectiveness of anti-TNF therapy, whereas no significant association was observed for vedolizumab or ustekinumab, may reflect differences in their mechanisms of action or insufficient statistical power to exclude a more modest effect. Unlike anti-TNF therapy, vedolizumab selectively inhibits lymphocyte trafficking to the intestinal mucosa, whereas ustekinumab targets the IL-12/23 pathway, mechanisms that may be less susceptible to the systemic pro-inflammatory effects of cigarette smoking. However, given the relatively small number of patients receiving these therapies, these findings should be interpreted with caution. In addition, these therapies were introduced during different therapeutic eras and were often prescribed at different stages of disease progression. Therefore, it remains unclear whether the observed differences reflect true biological mechanisms or simply limited statistical power. Confirmation in larger prospective studies is needed.

One of the most noteworthy findings of our study is that the negative association between smoking and anti-TNF effectiveness was observed not only in Crohn’s disease but also in ulcerative colitis. At first glance, this finding appears to contrast with the well-established epidemiological observation that cigarette smoking reduces the risk of developing UC and, in some studies, has been associated with a milder disease course. However, these observations primarily relate to disease susceptibility rather than to the response to targeted therapies once chronic intestinal inflammation has become established. Our findings therefore suggest that the apparent protective effect of smoking in UC should not be extrapolated to patients receiving biologic therapy. Instead, active smoking may represent a negative predictor of treatment effectiveness in UC, emphasizing that disease susceptibility and therapeutic responsiveness are biologically distinct processes. Although the association in UC was statistically significant despite the small number of smokers in this subgroup (27 conventional cigarette smokers and 7 e-cigarette users among 136 patients), larger multicenter studies are needed to confirm this finding and assess its potential implications for the therapeutic management of UC.

An additional clinically relevant finding was that active smokers more frequently required treatment sequencing than never smokers despite a comparable disease duration and duration of follow-up. Treatment sequencing is increasingly recognized as a surrogate marker of difficult-to-treat disease and is associated with higher healthcare costs, greater cumulative exposure to immunosuppressive therapies, and potentially poorer long-term outcomes. This issue was recently investigated by Alizadeh and colleagues in 172 patients with CD [33]. No significant association with smoking was observed in that study; however, only 13% of patients were active smokers. Although causality cannot be inferred from our retrospective study, our findings suggest that smoking may contribute not only to reduced effectiveness of anti-TNF therapy but also to an earlier need for therapeutic switching during the disease course.

From a practical perspective, these findings further reinforce current recommendations advocating smoking cessation in all patients with IBD. Traditionally, smoking cessation has been recommended primarily because of its detrimental effects on Crohn’s disease progression and its well-established systemic consequences. Our results provide an additional rationale by suggesting that smoking may also compromise the effectiveness of advanced therapies. Smoking status should therefore be systematically assessed before initiating biologic therapy and regularly reassessed during follow-up. Patients who continue smoking while receiving anti-TNF therapy may warrant closer clinical monitoring, earlier assessment of treatment effectiveness, and, where available, proactive therapeutic drug monitoring. Former smokers in our cohort achieved treatment outcomes comparable to those of never smokers. Whether smoking cessation after initiation of biologic therapy restores therapeutic responsiveness remains an important unanswered question that warrants prospective investigation. Likewise, future studies should evaluate the impact of e-cigarettes and heated tobacco products on treatment outcomes.

Our study has several strengths. It includes one of the largest single-center cohorts evaluating the relationship between smoking and the effectiveness of advanced therapies in IBD, with detailed characterization of smoking habits, a mean follow-up exceeding 14 years, and evaluation of more than 600 treatment courses across multiple therapeutic classes. The inclusion of both Crohn’s disease and ulcerative colitis further enhances the clinical relevance of our findings.

Several limitations should also be acknowledged. The retrospective single-center design limits the generalizability of our findings and precludes definitive conclusions regarding causality. In addition, residual confounding related to disease severity and treatment selection cannot be completely excluded despite adjustment for relevant clinical variables. The relatively small number of patients receiving newer biologics and small molecules reduced the statistical power to detect treatment-specific differences. Smoking exposure was based on patient-reported information. Although smoking status was updated at least twice yearly during contemporary follow-up and was verified by telephone in 2026 when necessary, changes in smoking habits during the earlier years of this cohort, which extends back to 1999, may not have been captured with the same completeness. The cohort was predominantly Italian and Caucasian, which may also limit generalizability to more ethnically diverse populations. Because the study spans more than two decades, during which therapeutic options, treatment sequencing, monitoring strategies, and prescribing regulations changed substantially, calendar-period effects cannot be excluded. The potential influence of concomitant corticosteroid or immunomodulator therapy could not be adequately evaluated. Dose optimization was used in approximately 20–30% of patients but was not systematically tracked, preventing assessment of whether optimization strategies differed according to smoking status. Therapeutic drug monitoring and anti-drug antibody measurements were not available and therefore could not be incorporated into the analysis. Finally, smoking-cessation duration was available in only 26 of 76 former smokers, limiting conclusions regarding the timing and potential reversibility of smoking-associated effects.

Our findings suggest that cigarette smoking may represent a clinically relevant and potentially modifiable determinant of treatment effectiveness in IBD. In addition, multicenter prospective studies integrating clinical, pharmacokinetic, immunological, microbiome, and molecular biomarkers are warranted to validate these observations, clarify the underlying mechanisms, and determine whether smoking status should be incorporated into therapeutic stratification algorithms. Our findings further suggest that smoking should no longer be viewed solely as a modifier of disease susceptibility in IBD but also as a potentially modifiable determinant of treatment effectiveness once biologic therapy has been initiated. The observation that former smokers achieved outcomes comparable to never smokers is compatible with the possibility that the detrimental effect of smoking may be at least partially reversible; however, this interpretation remains tentative because cessation duration was available for only 26 of 76 former smokers.

## 5. Conclusions

Active smoking was associated with reduced effectiveness of anti-TNF therapy in patients with inflammatory bowel disease, affecting not only Crohn’s disease but, unexpectedly, also ulcerative colitis. Although no similar association was observed with vedolizumab or ustekinumab, the limited number of patients receiving these therapies precludes definitive conclusions regarding treatment class-specific effects. Larger prospective studies are needed to validate these findings, clarify the underlying biological mechanisms, and evaluate the impact of smoking on newer therapeutic classes, including IL-23 inhibitors and small molecules. Meanwhile, our findings provide additional support for systematic smoking cessation counseling as an integral component of routine IBD care. Smoking status should be regarded as a potentially modifiable determinant of treatment effectiveness, and patients should be informed that continued smoking may adversely affect the outcomes of anti-TNF therapy. If confirmed in prospective studies, smoking status may become a simple and readily available factor to support therapeutic stratification and treatment optimization in patients with IBD.

## Figures and Tables

**Figure 1:**
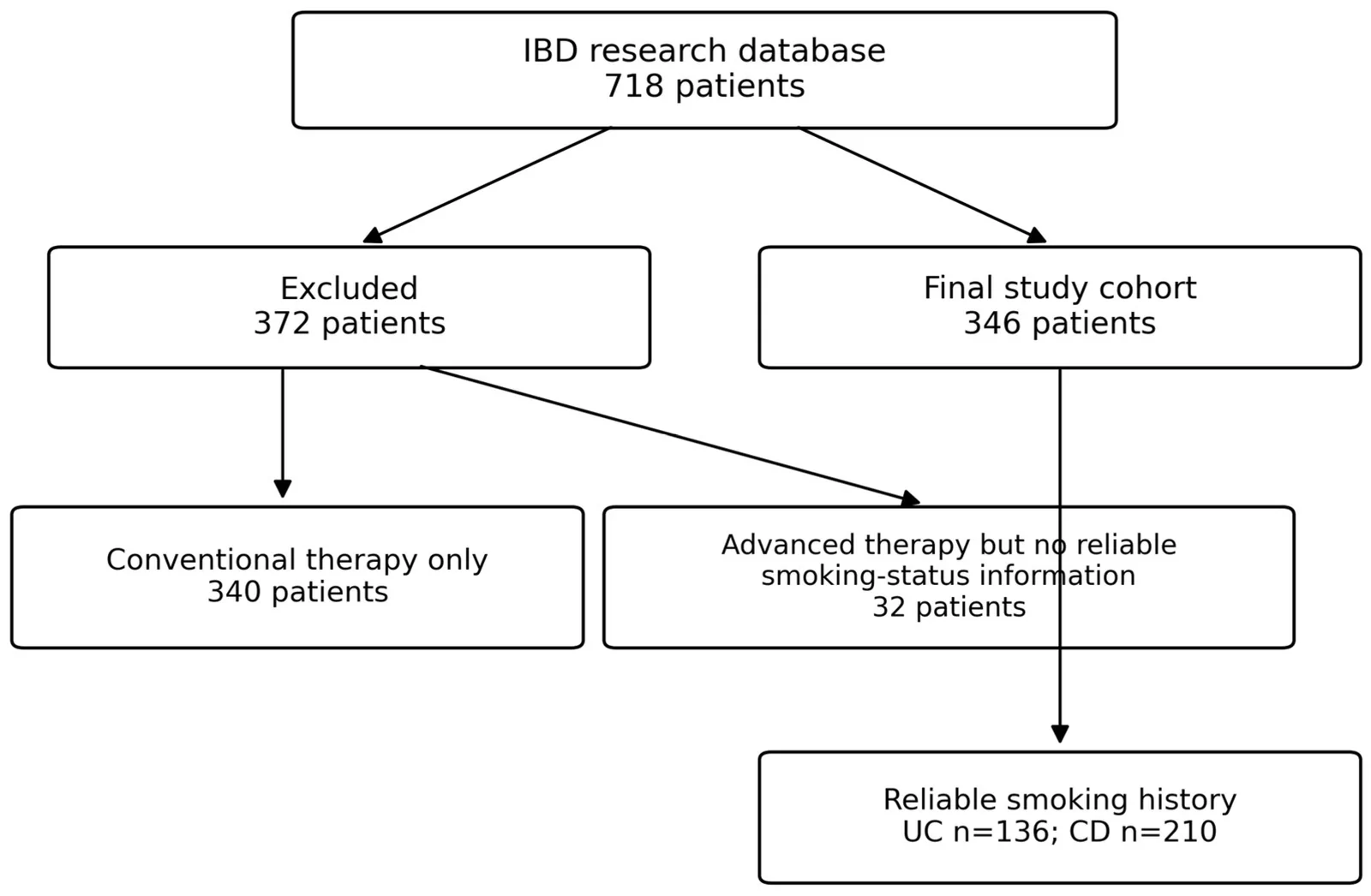
Patient inclusion flow diagram. The research database included 718 patients. A total of 372 patients were excluded: 340 had received conventional therapy only and 32 had received advanced therapy but lacked reliable smoking-status information. The final study cohort comprised 346 patients (136 UC and 210 CD).

**Table 1:** Main clinical characteristics of the study population (data given as mean ± SD and percentage).

Parameters	UC (*n* = 136)	CD (*n* = 210)	*p* Value
Age at diagnosis (yrs)	34.9 ± 14.5	32.4 ± 14.5	
Age at diagnosis (range)	7–70	7–77	
Age at last follow-up (yrs)	49 ± 16	46 ± 15.9	
Male/Female	74/62	116/94	
Smokers *n* (%)	34 (25.0)	73 (34.8)	=0.055
Former smokers *n* (%)	31 (22.8)	45 (21.4)	
E-Cig smokers *n* (%)	Included with smokers	Included with smokers	
Never smokers *n* (%)	71 (52.2)	92 (43.8)	
UC Localization (E1/E2/E3)	24/56/56		
CD Localization (L1/L2/L3)		79/44/77	
L1 + L4/L2 + L4/L3 + L4		6/1/3	
Disease Behavior (B1/B2/B3)		117/61/32	
Perianal disease	5 (3.7)	50 (23.8)	<0.0001
Abdominal surgery	14 (10.3)	85 (40.4)	<0.0001
EIM ^1^	42 (30.9)	80 (38)	

^1^ Extraintestinal manifestations.

**Table 2:** Advanced therapy (AT) for IBD in the study population.

Therapy	UC (*n* = 136)	CD (*n* = 210)
Infliximab	94	129
Adalimumab	35	113
Golimumab	10	1 *
Vedolizumab	52	40
Ustekinumab	19	65
JAKs	29	7
IL-23 inhibitors	6	10
Etrasimod	4	-
**Courses of AT** (*n* = 614)		
1	71	112
2	27	57
3	29	30
4	8	6
5	1	5

* Given because of rheumatologic indication.

**Table 3:** Breakdown of the effectiveness of advanced therapy on symptoms in IBD.

Drugs	Intolerance	Remission/ Response	Failure	Loss of Response
Infliximab (*n* = 223)	44 (19.7%)	118 (52.9%)	36 (16.1%)	25 (11.2%)
Adalimumab (*n* = 148)	10 (6.7%)	79 (53.4%)	38 (25.6%)	21 (14.2%)
Golimumab (*n* = 11)	0	1 (9%)	8 (72.7%)	2 (18.2%)
Vedolizumab (*n* = 92)	2 (2.2%)	55 (59.8%)	24 (26.1%)	11 (11.9%)
Ustekinumab (*n* = 84)	0	56 (66.7%)	12 (14.3%)	16 (19.1%)
JAKs (*n* = 36)	2 (5.5%)	29 (80.5%)	4 (11.1%)	1 (2.8%)
IL-23 blockers (*n* = 16)	0	15 (93.7%)	0	1 (6.2%)
Etrasimod (*n* = 4)	0	4 (100%)	0	0

**Table 4:** Correlation of smoking pattern with the effectiveness of all advanced therapies (**a**), anti-TNF agents (**b**), vedolizumab and ustekinumab (**c**).

(**a**)										
	**All AT** (*n* = 614)		**Intol** (*n* = 58)		**Resp** (*n* = 357)		**Fail** (*n* = 122)		**LOR** (*n* = 77)	
	**UC**	**CD**	**UC**	**CD**	**UC**	**CD**	**UC**	**CD**	**UC**	**CD**
**Smokers**	63	132	5	16	**30**	**65**	**13**	**24**	**15**	**27**
**Former**	58	77	4	11	36	49	15	14	3	3
**Never**	132	152	9	13	**79**	**98**	**34**	**22**	**10**	**19**
(**b**)										
	**IFX** (*n* = 223)		**Intol** (*n* = 44)		**Resp** (*n* = 118)		**Fail** (*n* = 36)		**LOR** (*n* = 25)	
	**UC** (*n* = 94)	**CD** (*n* = 129)	**UC**	**CD**	**UC**	**CD**	**UC**	**CD**	**UC**	**CD**
**Smokers** (*n* = 61)	23	38	5	11	**8**	**17**	**5**	**5**	**5**	**5**
Former (*n* = 52)	24	28	3	6	13	17	7	3	1	2
**Never** (*n* = 110)	47	63	9	10	**24**	**39**	**10**	**6**	**4**	**8**
	**ADA** (*n* = 148)		**Intol** (*n* = 10)		**Resp** (*n* = 79)		**Fail** (*n* = 38)		**LOR** (*n* = 21)	
	**UC** (*n* = 35)	**CD** (*n* = 113)	**UC**	**CD**	**UC**	**CD**	**UC**	**CD**	**UC**	**CD**
**Smokers** (*n* = 51)	7	44	0	2	**1**	**22**	**5**	**9**	**1**	**11**
**Former** (*n* = 27)	3	24	1	3	1	13	1	7	0	1
**Never** (*n* = 70)	25	45	0	4	**13**	**29**	**10**	**6**	**2**	**6**
(**c**)										
	**Vedolizumab** (*n* = 92)		**Intol** (*n* = 2)		**Resp** (*n* = 55)		**Fail** (*n* = 24)		**LOR** (*n* = 11)	
	**UC** (*n* = 52)	**CD** (*n* = 40)	**UC**	**CD**	**UC**	**CD**	**UC**	**CD**	**UC**	**CD**
**Smokers** (*n* = 30)	18	12	--	--	9	10	2	2	7	--
**Former** (*n* = 18)	8	10	0	2	4	6	4	2	--	--
**Never** (*n* = 44)	26	18	--	--	17	9	7	7	2	2
	**Ustekinumab** (*n* = 84)		**Intol** (*n* = 0)		**Resp** (*n* = 56)		**Fail** (*n* = 12)		**LOR** (*n* = 16)	
	**UC** (*n* = 19)	**CD** (*n* = 65)	**UC**	**CD**	**UC**	**CD**	**UC**	**CD**	**UC**	**CD**
**Smokers** (*n* = 41)	4	37	--	--	3	21	0	6	1	10
**Former** (*n* = 9)	3	6	--	--	3	4	0	2	--	--
**Never** (*n* = 34)	12	22	--	--	9	16	1	3	2	3

AT = advanced therapy; Intol = intolerance; Resp = responder; Fail = failure; LOR = loss of response. **Significant differences in bold** for responders versus failure and LOR among smokers and never smokers (*p* values in the text). Smokers and e-cigarette smokers are pooled together. IFX = infliximab; ADA = adalimumab. **Significant differences in bold** for responders versus failure and LOR among smokers and never smokers (*p* values in the text). Golimumab (*n* = 11) was excluded from the anti-TNF effectiveness analysis because of the small sample size and atypically high primary-failure rate.

**Table 5:** Correlation of smoking with need for additional courses of advanced therapy.

	Smokers	Former Smokers	Never Smokers
**UC** (*n* = 136)	(*n* = 34)	(*n* = 31)	(*n* = 71)
Age at diagnosis (yrs)	34.8 ± 10	36.2 ± 17.1 (*p* = 0.68 vs. smokers)	33.9 ± 14.5 (*p* = 0.74 vs. smokers)
Disease duration (yrs)	16.9 ± 10.8	14.1 ± 11.3 (*p* = 0.30 vs. smokers)	13.3 ± 9.9 (*p* = 0.09 vs. smokers)
Duration of f-up (yrs)	11.4 ± 8.9	10.4 ± 9.5 (*p* = 0.66 vs. smokers)	10.2 ± 8.7 (*p* = 0.51 vs. smokers)
Surgery	2 (5.8%)	3 (9.6%)	9 (12.6%)
1 Course AT	9 (26.5%)	13 (41.9%)	34 (47.8%)
≥2 Courses	25 (73.5%) *	18 (58.1%)	37 (52.2%)
**CD** (*n* = 210)	(*n* = 73)	(*n* = 45)	(*n* = 92)
Age at diagnosis (yrs)	33.8 ± 13.6	33.8 ± 15.1 (*p* = 0.99 vs. smokers)	30.4 ± 15.2 (*p* = 0.27 vs. smokers)
Disease duration (yrs)	15.4 ± 11	13.5 ± 11.2 (*p* = 0.49 vs. smokers)	13.1 ± 9.2 (*p* = 0.26 vs. smokers)
Duration of f-up (yrs)	12.3 ± 9.1	11.3 ± 8.5 (*p* = 0.64 vs. smokers)	10.5 ± 8.5 (*p* = 0.32 vs. smokers)
Surgery	33 (45.2%) #	22 (48.9%) #	30 (32.6%)
1 Course AT	25 (34.2%)	25 (55.5%)	47 (51.1%)
≥2 Courses	48 (65.8%) *	20 (44.5%)	45 (48.9%)

* *p* = 0.03 vs. never smokers; # *p* = 0.04 vs. never smokers. Smokers and e-cig smokers are pooled together.

**Table 6:** Adjusted multivariable logistic regression and mixed-effects logistic regression including patient identifier as a random effect. Odds ratios > 1 indicate a higher likelihood of treatment response in never smokers compared with active smokers. The adjusted models included therapy type, line of therapy, disease duration, disease type, and surgery as covariates.

Model/Variable	Beta Coefficient (β)	Standard Error (SE)	Z-Score	Odds Ratio (OR)	95% CI	*p* Value
All AT (Adjusted)	0.223	0.109	2.054	1.25	1.01–1.55	0.04
All AT (Mixed effect)	0.095	0.053	1.812	1.10	0.99–1.22	0.07
Anti-TNF (Adjusted)	0.365	0.168	2.170	1.44	1.04–2.00	0.03
Anti-TNF (Mixed effect)	0.300	0.146	2.054	1.35	1.01–1.80	0.04

## Data Availability

The research dataset may be requested from the authors for bona fide collaborative research, subject to applicable ethical and data-protection requirements.

## Abbreviations

The following abbreviations are used in this manuscript:

ADA	Adalimumab
AT	Advanced therapy
CD	Crohn’s disease
CDAI	Crohn’s disease activity index
CI	Confidence interval
CRC	Colorectal cancer
FC	Fecal calprotectin
EIM	Extraintestinal manifestation
HBI	Harvey Bradshaw index
HR	Hazard ratio
IBD	Inflammatory bowel disease
IFX	Infliximab
IRCCS	Istituto Ricerca e Cura a Carattere Scientifico (Research and Care Scientific Institute)
LOR	Loss of response
pMAYO	Partial MAYO score
OR	Odds Ratio
RCT	Randomized controlled trial
RR	Relative Risk
SES-CD	Simple Endoscopic Score for Crohn’s Disease
STRIDE	Selecting Therapeutic Targets in Inflammatory Bowel Disease (Consensus)
TNF	Tumor necrosis factor
UC	Ulcerative colitis
JAK	Janus Kinase
HNBT	Heat-not-burn tobacco
NF-κB	Nuclear factor kappa B
